# Using CRISPR-Kill for organ specific cell elimination by cleavage of tandem repeats

**DOI:** 10.1038/s41467-022-29130-w

**Published:** 2022-03-21

**Authors:** Angelina Schindele, Fabienne Gehrke, Carla Schmidt, Sarah Röhrig, Annika Dorn, Holger Puchta

**Affiliations:** Botanical Institute – Molecular Biology and Biochemistry of Plants, Karlsruhe Institute of Technology, Fritz-Haber-Weg 4, 76131 Karlsruhe, Germany

**Keywords:** Tissue engineering, Molecular engineering in plants, Plant development

## Abstract

CRISPR/Cas has been mainly used for mutagenesis through the induction of double strand breaks (DSBs) within unique protein-coding genes. Using the SaCas9 nuclease to induce multiple DSBs in functional repetitive DNA of *Arabidopsis thaliana*, we can now show that cell death can be induced in a controlled way. This approach, named CRISPR-Kill, can be used as tool for tissue engineering. By simply exchanging the constitutive promoter of SaCas9 with cell type-specific promoters, it is possible to block organogenesis in *Arabidopsis*. By AP1-specific expression of CRISPR-Kill, we are able to restore the *apetala1* phenotype and to specifically eliminate petals. In addition, by expressing CRISPR-Kill in root-specific pericycle cells, we are able to dramatically reduce the number and the length of lateral roots. In the future, the application of CRISPR-Kill may not only help to control development but could also be used to change the biochemical properties of plants.

## Abstract (web summary)

How double strand breaks (DSBs) are repaired within the plant 45S rDNA repeats is unclear. Here, the authors show that Cas9-mediated DSBs in 45S rDNA are mainly repaired by cNHEJ and describe CRISPR-Kill as a tool for organ-specific cell elimination by targeting functional repetitive DNA in Arabidopsis.

## Introduction

Since its first description as programmable nuclease^1^, the use of Cas9 has revolutionized biology. In plants, CRISPR/Cas systems are now widely used for genome editing in basic science as well as for trait improvement in breeding^2–5^. Beside that, CRISPR/Cas can also be used to study DSB repair itself. Multiple studies on DSB repair in unique genomic sequences have been performed but very little is known on how repeats are repaired within repetitive sequences^6^.

The ribosomal RNA genes (rDNA) of *Arabidopsis thaliana* are arranged in up to 750 10-kb-long tandem repeats per haploid genome^7,8^. Each repeat consists of the 18S, 5.8S, and 25S rRNA genes, separated by two conserved internal transcribed spacers (ITS1 and ITS2) and a variable intergenic spacer (IGS).

DSBs can be repaired by two pathways: non-homologous end-joining (NHEJ) or homologous recombination (HR)^9^. In somatic plant cells, the error-prone NHEJ pathway is the prevalent repair mechanism, which can be subdivided into the KU-heterodimer- and LIG4-dependent classical NHEJ (cNHEJ) pathway and the POLQ-dependent microhomology-mediated end-joining (MMEJ) pathway. Whereas cNHEJ mediates the direct ligation of the broken ends, often resulting in smaller insertions and deletions, MMEJ utilizes microhomologies (MH) close to the break sites, resulting in a loss of sequence between the MH^10,11^. Repair via HR requires a template and, in the case of tandem repeats, might result in repeat loss. Recently, it was shown that HR is downregulated within the 45S rDNA during meiosis, and that DSBs within this region are repaired via classical NHEJ (cNHEJ)^12^. However, it has been shown that HR plays a major role in the somatic repeat loss of 45S rDNA in the *caf-1* mutant background in *Arabidopsis*^13^. To clarify the situation, the initial goal of this study was to directly induce DSBs within the 45S rDNA repeats by Cas9 to define by which pathway they are preferentially repaired. Here, we show that Cas9-mediated DSBs in 45S rDNA are mainly repaired by cNHEJ and furthermore describe a system for the controlled induction of cell death by targeting functional repetitive DNA.

## Results

### DSB repair in the variable region of the 45S rDNA

**Fig. 1:**
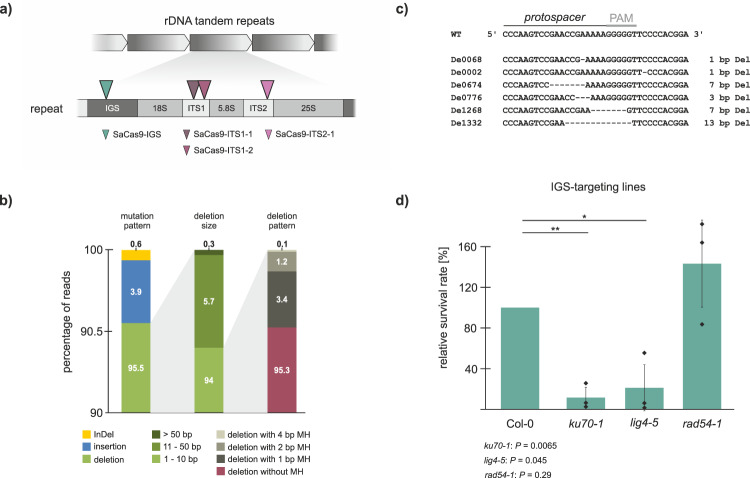
Cleavage of the IGS region of the 45S rDNA repeats in *Arabidopsis* using SaCas9. **a** The 45S rDNA in *Arabidopsis* is arranged in up to 750 repeats, in a head-to-tail-fashion. Each repeat consists of a unit of three rRNA genes (18S, 5.8S, and 25S), separated by a variable intergenic spacer (IGS) region. The individual rRNA genes are separated by two highly conserved internal transcribed spacers (ITS). The triangles represent the cleavage sites for SaCas9 within the repeats. By targeting the variable IGS region (petrol), only a subset of repeats was cleaved. **b** The break site was analyzed by deep sequencing. Only a very small fraction of mutations was identified as InDels (yellow) or insertions (blue), whereas over 95% were deletions (green). Further analysis of the reads, including the deletions, showed that 94% of these were small deletions with a size of 10 bp or smaller. The deletion pattern of these small deletions revealed that no microhomologies were used for the repair (red). **c** Detailed illustration of exemplary junctions with deletions in Col-0 plants on the sequence level. **d** Cleavage of the IGS region in DNA repair mutants revealed that only the cNHEJ mutants, *ku70-1* and *lig4-5*, but not the HR mutant, *rad54-1*, responded highly sensitive, resulting in a drastically reduced survival rate (*n* = 3 biologically independent experiments). Data are presented as normalized mean values ± SD. Statistical differences were calculated using the two-tailed *t*-test with unequal variances: *P < 0.05, **P < 0.01, ***P < 0.001, ****P < 0.0001. Source data are provided as a Source data file.

To determine the mechanism of DSB repair in the 45S rDNA in somatic cells, we induced DSBs within the 45S rDNA repeats using *Staphylococcus aureus* Cas9 (*Sa*Cas9)^14^. The target site was selected in the variable IGS region, so that only a subset of repeats was cleaved (Fig. 1a; Supplementary Table 1). As a control, we designed a gRNA targeting the non-essential *ADH1* gene (Ubi-SaCas9-ADH1) which induces a unique DSB. Col-0 plants were transformed with the expression vector, Ubi-SaCas9-IGS, as well as the control vector. After two weeks of growth, 40 transgenic plants per line were selected and pooled for extraction of genomic DNA. Subsequently, the 45S rDNA target site was amplified and subjected to next-generation sequencing (NGS) to determine the repair pattern. The deep sequencing analysis revealed that the majority of mutations at the break site were deletions, most of them smaller than 10 bp, and that the repair occurred without MH (Fig. 1b and c). Only a tiny fraction of reads showed insertions or InDels at the break site. These results strongly imply that the cNHEJ is the major DSB repair pathway within the 45S rDNA. To verify this, DSBs were induced in the 45S rDNA of DNA repair mutants at the same target site. The cNHEJ mutants, *ku70-1* and *lig4-5*, as well as the HR mutant, *rad54-1*, were transformed with the expression and the control construct. The RAD54 mutant was chosen because it is, in contrast to other HR factor mutants, not sterile but has a strong deficiency in somatic HR^15^. After two weeks of growth, the survival rates of all transformed mutant lines were determined and normalized to Col-0. We expected that an insufficient 45S rDNA repair, due to loss of DNA repair factors, should result in a reduction of the survival rate. Indeed, both cNHEJ mutant lines, *ku70-1* and *lig4-5*, showed a dramatic reduction in survival rate, when the 45S rDNA was cleaved (Fig. 1d). In contrast, no reduction of the survival rate could be observed in the *rad54-1* HR mutant line. Thus, cNHEJ plays a key role in 45S rDNA DSB repair in somatic cells.

### DSB induction in the conserved region of the 45S rDNA

**Fig. 2:**
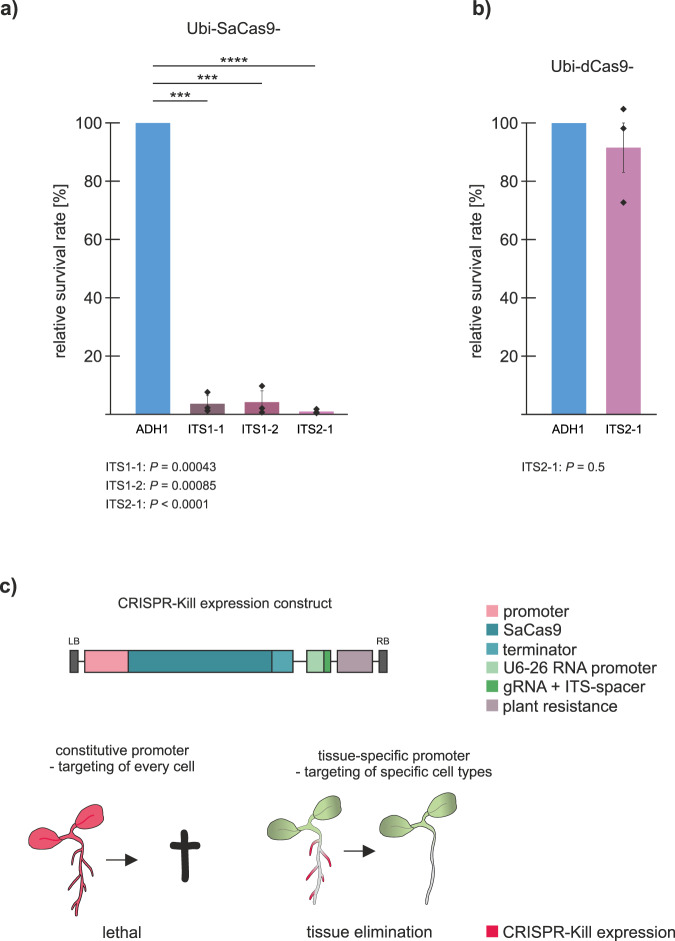
Cleavage of the ITS region of the 45S rDNA repeats in *Arabidopsis* using SaCas9. **a** Cleavage of the conserved ITS region of the 45S rDNA using SaCas9 in the wild type resulted in a dramatic decrease of the number of transformants in all three ITS-targeting lines (Ubi-SaCas9-ITS1-1, Ubi-SaCas9-ITS1-2, and Ubi-SaCas9-ITS2-1) compared to the control line, Ubi-SaCas9-ADH1 (*n* = 3 biologically independent experiments). Data are presented as normalized mean values ± SD. *P* = 0.00043 for ITS1-1; *P* = 0.00085 for ITS1-2; *P* < 0.0001 for ITS2-1. **b** The survival rate of lines transformed with a catalytically inactive SaCas9 (dCas9) targeting ADH1 (Ubi-dCas9-ADH1) and ITS2 (Ubi-dCas9-ITS2-1) was analyzed. No reduction in survival rate could be observed in the ITS2-1 line compared to the ADH1 line (*n* = 3 biologically independent experiments). Data are presented as normalized mean values ± SD. **c** SaCas9 (blue) is under the expression of an exchangeable promoter (light pink). Utilization of SaCas9 under expression of a constitutive results in cleavage of the 45S rDNA in all cells (red), leading to cell death. By exchanging the constitutive promoter with a tissue-specific promoter, it is possible to obtain plants devoid of individual organs. Statistical differences were calculated using the two-tailed *t*-test with unequal variances: *P < 0.05, **P < 0.01, ***P < 0.001, ****P < 0.0001. Source data are provided as a Source data file.

Next, we used the two intergenic transcribed spacer (ITS) regions as target sites, which are conserved between all repeats. Two gRNAs were designed for ITS1 (Ubi-SaCas9-ITS1-1 and Ubi-SaCas9-ITS1-2) and one guide for ITS2 (Ubi-SaCas9-ITS2-1) (Fig. 1a; Supplementary Table 1). Again, the gRNA targeting the non-essential *ADH1* gene (Ubi-SaCas9-ADH1) was used as a control, which showed an editing efficiency of 60% (Supplementary Fig. 1a). Transformation with the T-DNA targeting *ADH1* proved to be efficient: 3.2% of the progeny were gentamycin-resistant. In contrast, a dramatic decrease in transformants was observed in the ITS target lines, with a reduction of 96% in case of Ubi-SaCas9-ITS1-1 as well as Ubi-SaCas9-ITS1-2 and a reduction of 99% in case of Ubi-SaCas9-ITS2-1 (Fig. 2a). The fact that quantitative real-time PCR showed no alteration in 45S rDNA copy number in the surviving seedlings suggests that in these few cases, the T-DNA was either truncated or silenced, resulting in insufficient DSB induction (Supplementary Fig. 1b and c). To prove that the nuclease activity of the SaCas9, and not just simple binding of the protein to the rDNA, is required, an additional set of control lines was transformed with catalytically inactive SaCas9 (dCas9) targeting ADH1 (Ubi-dCas9-ADH1) and ITS2 (Ubi-dCas9-ITS2-1). The survival rate determined in both dCas9 approaches was similar and comparable to that of the Ubi-SaCas9-ADH1 control line (Fig. 2b). Thus, the sgRNA ITS2-1 was only able to induce a lethal effect in combination with an active nuclease, demonstrating that massive DSB induction is responsible for the lethal effect. This result was surprising, considering that a recent study reported a dramatic reduction of copy number, but not lethality, when another Cas9 orthologue, namely *Streptococcus pyogenes* Cas9 (SpCas9), was used for DSB induction^16^. This difference might have been caused by the fact that SaCas9 is superior in DSB induction in comparison to SpCas9 in *Arabidopsis*^14,17^. Our results clearly demonstrate that targeting the conserved part of the 45S rDNA with a highly efficient nuclease is lethal for the transformed cells (Fig. 2c). Based on this observation, we speculated that massive DSB induction could be used as a tool to induce cell death during plant development. Thus, if Cas9 expression was confined to specific growth phases or specific organs, inducing cell death in a highly specific and cell-autonomous manner, a novel form of tissue engineering could be developed (Fig. 2c). By using this system, named CRISPR-Kill, we expected to be able to obtain plants devoid of individual organs or cell types.

### CRISPR-Kill for elimination of floral organs

**Fig. 3:**
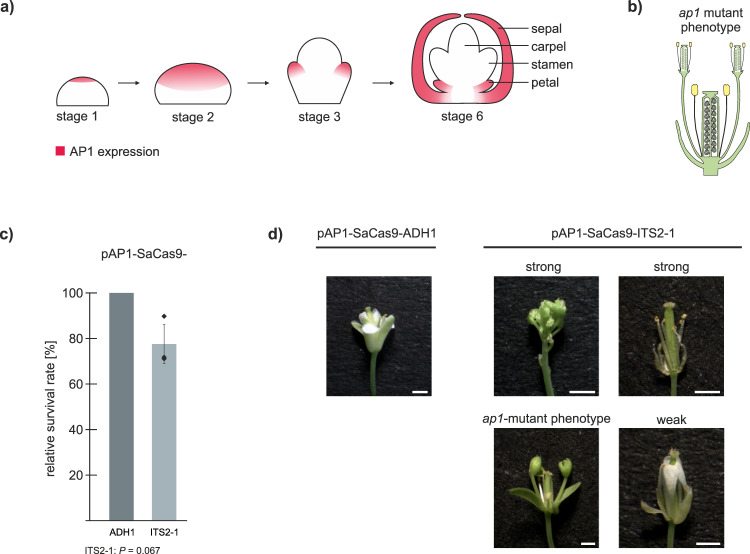
CRISPR-Kill-mediated floral tissue elimination by cleaving 45S rDNA repeats in *Arabidopsis*. **a** The floral homeotic protein, APETALA1 (AP1), is highly expressed in the early floral meristem at flower development stages 1 and 2 (red). At development stage 3, the expression is restricted to specific tissue, which is determined to form sepals and petals. **b** Schematic representation of the *ap1* mutant phenotype: a disturbed flower architecture with an extra set of carpel and stamen instead of sepals and petals. **c** A comparison of survival rates between the T1 control line (pAP1-SaCas9-ADH1) and the T1 flower-specific CRISPR-Kill line (pAP1-SaCas9-ITS2-1) revealed no significant differences (*n* = 3 biologically independent experiments). Data are presented as normalized mean values ± SD. Statistical differences were calculated using the two-tailed *t*-test with unequal variances. **d** Representative floral phenotypes of pAP1-SaCas9-ADH1 control line and pAP1-SaCas9-ITS2-1 CRISPR-Kill line. Bar = 1 mm. Statistical differences were calculated using the two-tailed *t*-test with unequal variances. Source data are provided as a Source data file.

**Fig. 4:**
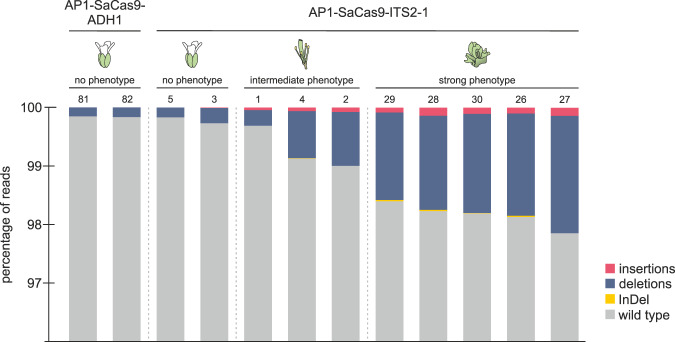
Deep sequencing of floral CRISPR-Kill lines. The target site of ITS2-1 was analyzed via deep sequencing in floral tissue of two ADH1 control lines (#81 and #82) and ten ITS2-1 lines. The CRISPR-Kill lines were subdivided according to phenotype in ‘no phenotype’ (#5 and #3), ‘intermediate phenotype’ (#1, #4, and #2), and ‘strong phenotype’ (#26 to #30). The reads of the two ADH1 lines showed no repair events (gray), only also naturally occurring 1 bp-deletions (blue). In contrast, analysis of the CRISPR-Kill lines with a distinguishable phenotype revealed repair events like deletions (blue), insertions (red), and InDels (yellow), at the break site, with mutation frequencies linked to the severity of the phenotype. Source data have been deposited in SRA [accession: PRJNA726366].

As a proof of concept, we decided to test Cas9 expression under control of the promoter of the floral homeotic gene *APETALA1* (*AP1*, At1g69120). AP1 is a key player for flower meristem identity. It is highly expressed at the early stages of flower development and later gets restricted to specific tissue where it is required for sepal and petal development^18^ (Fig. 3a). The *ap1* mutant shows a disturbed flower architecture where, instead of petals, carpel and stamen are formed (Fig. 3b). We were eager to test if the use of CRISPR-Kill would enable us to specifically eliminate petal tissue. For this approach, the most efficient guide, ITS2-1, was chosen, while the *ADH1* guide served as a control. We expected that the utilization of the *AP1* promoter would restrict SaCas9 expression exclusively to the early floral meristem and, later on, to sepals and petals. After determination of the transformation rate, no significant reduction could be observed in pAP1-SaCas9-ITS2-1 in comparison to pAP1-SaCas9-ADH1 T1 plants (Fig. 3c). After 4 weeks, 133 independent pAP1-SaCas9-ITS2-1 T1 plants were analyzed for phenotypical deviations in comparison to the control lines. While the control plants showed normal growth, a spectrum of different phenotypes was observed for the CRISPR-Kill plants (Table 1 and Fig. 3d). A very small fraction of CRISPR-Kill T1 lines had died, while one-fifth showed a strong phenotype, characterized by a lack of flowers or lack of sepals and petals. Another fifth showed a weak phenotype, characterized by a full set of sepals, but an incomplete set of petals. In almost half of all lines, an intermediate phenotype could be observed, described by the presence of, at least some, sepals in combination with varying numbers of petals. This intermediate phenotype also consists of the *ap1* mutant phenotype (19.8%), characterized by a disturbed floral architecture. To determine whether there is a correlation between the strength of the phenotype and the amount of induced DSBs in the respective line, we isolated floral tissue and performed deep sequencing analysis of the ITS2 target site. In total, two ADH1 control lines (#81 and #82), as well as ten ITS-2-1 lines were analyzed for InDels. The latter ones were subdivided based on their phenotype in ‘no phenotype’ (#5 and #3), ‘intermediate phenotype’ (#1, #4, and #2), and ‘strong phenotype’ (#26 to #30) (Fig. 4). Due to the fact that only a minor portion of respective cell types survived elimination and DNA from adjacent tissues was co-purified, we expected to detect InDels as indications of DSB repair in only a small fraction of reads. The reads of the two control lines showed no InDels at the break site. Occasionally, 1 bp deletions (0.15% for #81 and 0.16% for #82) could be observed, reflecting the natural variances within the repeats. In contrast, all CRISPR-Kill lines that showed a distinguishable phenotype revealed InDels at the break site, with mutation frequencies proportional to the severeness of the phenotype. In the CRISPR-Kill lines with the ‘intermediate phenotype’ mutation, frequencies between 0.31% and 0.99% could be observed. In turn, the CRISPR-Kill lines with the ‘strong phenotype’ showed mutation frequencies between 1.60% and 2.14% (Supplementary Figs. 2 and 3). These findings demonstrate that efficient organ elimination can be achieved by using the CRISPR-Kill system and that the strength of the phenotype is correlated with the efficiency of DSB induction.

**Table 1:** T1 phenotypes of CRISPR-Kill-mediated floral tissue elimination by cleaving 45S rDNA repeats.

	Phenotype	pAP1-SaCas9-ADH1	pAP1-SaCas9-ITS2-1
Lethal		0	1.5
Strong		0	19.8
	No flower	0	4.6
	No sepals, no petals	0	15.3
Intermediate		0	46.6
	Some sepals, no petals	0	7.6
	All sepals, no petals	0	7.6
	*ap1-*mutant phenotype	0	19.8
	Some sepals, some petals	0	11.5
Weak	All sepals, some petals	0	20.6
None	Wild type	100	11.5

Phenotyping of 4-week-old plants of the pAP1-CRISPR-Kill line. The different phenotypes were categorized into ‘lethal’, ‘strong’, ‘intermediate’, ‘weak’, and no phenotypical deviation compared to the control line. Nearly 20% of the analyzed pAP1-SaCas9-ITS2-1 lines showed a strong deformation of the floral tissue. Almost half of all lines showed an intermediate phenotype with different combinations of missing sepals and petals. The intermediate category also includes the *ap1* mutant phenotype (nearly 20%). Another fifth showed weak phenotypical differences compared to the control line, while a tenth showed no phenotypical deviations at all (ADH1: *n* = 19, ITS2-1: *n* = 133).

### Extension of CRISPR-Kill to target centromeric repeats

**Fig. 5:**
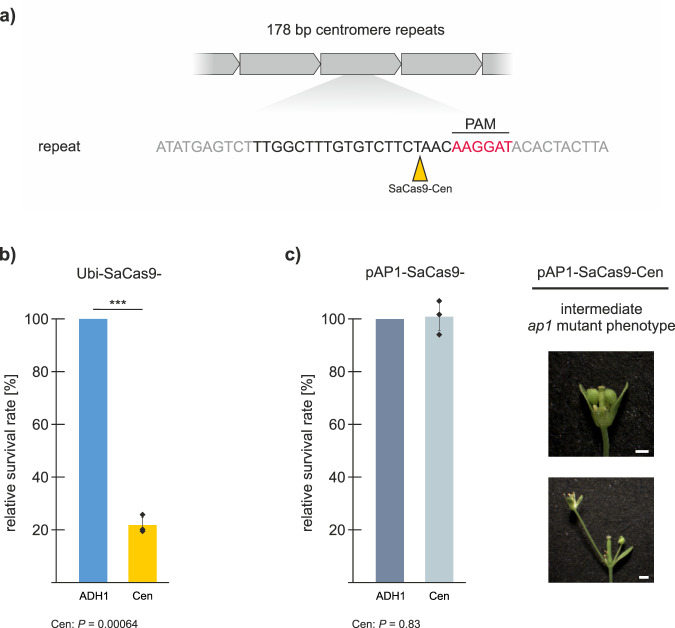
Establishment of a centromere-specific CRISPR-Kill system in *Arabidopsis*. **a** The core centromere is characterized by a highly repetitive cluster of functional tandem repeats. The yellow triangle represents the cleavage site for SaCas9 within the centromere repeats (SaCas9-Cen). **b** Transformation of Col-0 plants with Ubi-SaCas9-Cen resulted in a reduction of the survival rate of T1 plants by 80% (*n* = 3 biologically independent experiments). Data are presented as normalized mean values ± SD. *P* = 0.00064. **c** Floral-specific expression of SaCas9-Cen using the *AP1*-promoter did not result in a reduction of the survival rate (*n* = 3 biologically independent experiments). Data are presented as normalized mean values ± SD. The phenotypical analysis revealed an *ap1*-mutant phenotype with disturbed floral architecture in most cases. Bar = 1 mm. Statistical differences were calculated using the two-tailed *t*-test with unequal variances: ***P < 0.001. Source data are provided as a Source data file.

As the cleavage of 45S rDNA tandem repeats for tissue elimination was highly efficient, we speculated whether the cleavage of other repetitive elements would lead to a comparable result. The 178 bp centromeric sequences represent another highly repetitive cluster of functional tandem repeats^19,20^. Therefore, the gRNA of the CRISPR-Kill system was programmed to target the conserved 178 bp repeat for DSB induction (Ubi-SaCas9-Cen) (Fig. 5a). Transformation of Col-0 with this construct resulted in a reduction of resistant seedlings by 80% in comparison to the ADH1 control (Fig. 5b). In contrast, AP1-specific expression (pAP1-SaCas9-Cen) did not reduce the number of transformed seedlings (Fig. 5c). The phenotypical analysis revealed the *ap1* mutant phenotype as the most abundant, confirming the successful organ elimination (Fig. 5c). Interestingly, in these 40 independent lines the phenotype was much more uniform than in pAP1-SaCas9-ITS2-1.

### CRISPR-Kill for elimination of lateral roots

**Fig. 6:**
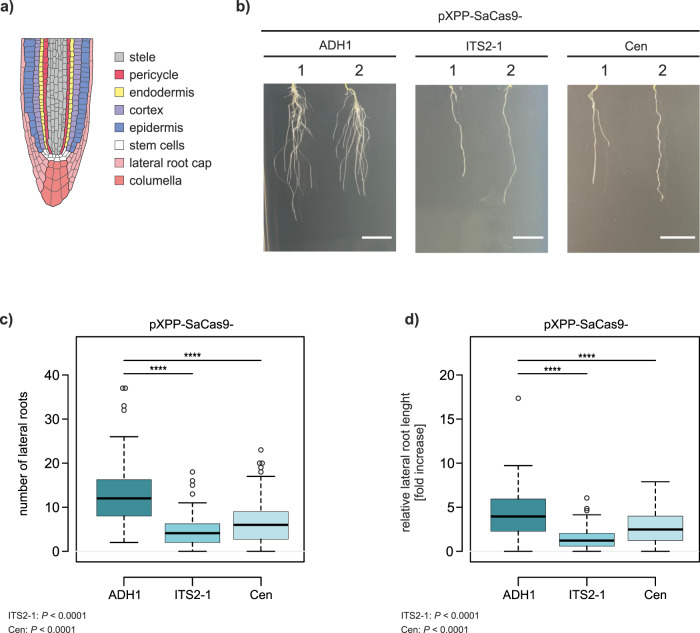
CRISPR-Kill-mediated lateral root elimination by cleavage of 45S rDNA and centromeric repeats. **a** Schematic overview of different root cell types in the *A. thaliana* root tip. The root tip consists of the lateral root cap and columella (pink). The central cells of the root (stele, gray) are framed by pericycle cells (red), followed by endodermis (yellow), cortex (purple), and epidermis (blue), which arise from the stem cell niche (white). Lateral roots arise from the pericycle cells (red). **b** Representative pictures of lateral roots in both CRISPR-Kill lines (pXPP-SaCas9-ITS2-1 and pXPP-SaCas9-Cen) in comparison to the control line (pXPP-SaCas9-ADH1). Bar = 1 cm. **c** Total number of lateral roots in control line and CRISPR-Kill lines. The control line, pXPP-SaCas9-ADH1, showed an average of 13 lateral roots per plant, whereas in the CRISPR-Kill lines an average of 5 (ITS2-1-CRISPR-Kill line) and 7 (Cen-CRISPR-Kill line) lateral roots per plant was observed, respectively. *P* < 0.0001 for ITS2-1; *P* < 0.0001 for Cen. **d** Lateral root length in relation to main root length. In the control line, the sum of the length of all lateral roots was on average 4.4 times longer than the main root. In contrast, the sum of the length of all lateral roots in the ITS2-1- and Cen-CRISPR-Kill lines was on average only 1.4 and 2.2 times longer than the main root, respectively. *P* < 0.0001 for ITS2-1; *P* < 0.0001 for Cen. Data are presented as box plots (*n* = 108), boxes show the first to third quartile with median, whiskers encompass 1.5x the interquartile range reaching to minimum and maximum, and data beyond that threshold is indicated as outliers. *P* values were calculated using the one-way ANOVA-test: ****P < 0.0001. Source data are provided as a Source data file.

**Fig. 7:**
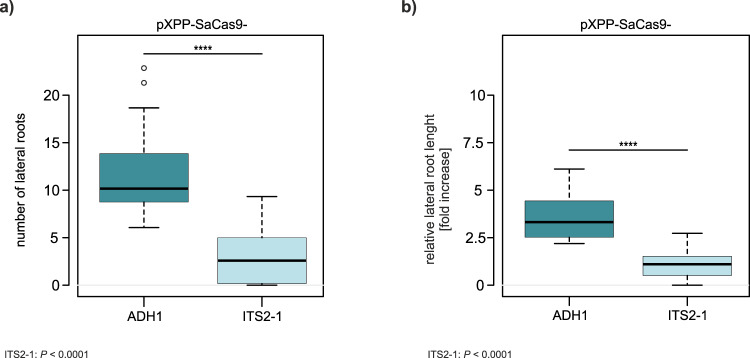
CRISPR-Kill-mediated lateral root elimination in T2 lines by cleavage of 45S rDNA. **a** Total number of lateral roots in control line and rDNA CRISPR-Kill lines. The control line, pXPP-SaCas9-ADH1, showed an average of 11 lateral roots per plant, whereas the ITS2-1-CRISPR-Kill line showed a 3-fold decrease and only an average of 3 lateral roots per plant. *P* < 0.0001. **b** Lateral root length in relation to main root length. A 3-fold decrease could also be observed in the relative lateral root length of the ITS2-1-CRISPR-Kill line. While the sum of the length of all lateral roots in the control line was on average 3.6 times longer than the main root, the sum of the length of all lateral roots in the ITS2-1 CRISPR-Kill line was on average only 1.1 times longer than the main root. *P* < 0.0001. Data is presented as box plots (*n* = 30), boxes show the first to third quartile with median, whiskers encompass 1.5x the interquartile range reaching to minimum and maximum, and data beyond that threshold is indicated as outliers. *P* values were calculated using the one-way ANOVA-test: ****P < 0.0001. Source data are provided as a Source data file.

To demonstrate the versatility of the system, we used CRISPR-Kill to modify root development. In *A. thaliana*, the pericycle cells form lateral roots (Fig. 6a). Thus, elimination of this cell type during root development was expected to hinder lateral root formation. Using the *XPP* (xylem pole pericycle) promoter, which is exclusively expressed in the pericycle^21,22^, we transformed Col-0 plants with XPP-SaCas9-ITS2-1, XPP-SaCas9-Cen constructs, and pXPP-SaCas9-ADH1 as a control (Supplementary Fig. 4a). Due to their early expression, a slight reduction of transformation efficiency could be observed in case of the two pXPP-CRISPR-Kill constructs compared to the control (Supplementary Fig. 4b). In all three approaches, 20-day old transgenic T1 plants were checked for lateral root growth. In the 45S rDNA CRISPR-Kill line, a three-fold decrease was observed in lateral root numbers, whereas the centromere CRISPR-Kill line showed a two-fold decrease (Fig. 6b and d). In addition, the relative lateral root length was determined (Supplementary Fig. 4c). Again, a three-fold decrease compared to the control line was observed in the rDNA-targeting line and a two-fold decrease in the centromere-targeting line (Fig. 6c and d). A detailed analysis of individual seedlings showed that, by using CRISPR-Kill, a larger portion of plants hardly developed any lateral roots (Supplementary Fig. 4d and e). This indicates that the severeness of the induced phenotype might vary depending on the T-DNA integration site, as seen in the case of pAP1-SaCas9-ITS2-1 in flower development. As we were interested in whether the phenotypical effects would be reproducible in the following generation, we analyzed root development in presence of XPP-SaCas9-ITS2-1 in the T2 generation. Progeny of T1 plants were selected for presence of the CRISPR-Kill construct and respective plants were analyzed as described above. For both lateral root number and relative lateral root length, again, a 3-fold decrease could be observed compared to the control (Fig. 7 and Supplementary Fig. 5).

## Discussion

Taken together, we were able to demonstrate that SaCas9-mediated induction of multiple DSBs in 45S rDNA and centromere repeats can be harnessed to trigger cell death in a cell-autonomous way, resulting in the highly efficient elimination of selected plant cell types. A broad spectrum of phenotypic outcomes was generated, indicating that this system might be a valuable tool to accomplish various stages of cell type or organ elimination. The CRISPR-Kill approach differs massively from others, which conditionally knock out genes in specific cell types to prevent pleiotropic effects or lethality^23,24^. In addition, it expands the method pool of traditional cell elimination approaches. The most common methods for cell elimination rely on the tissue-specific expression of bacterial toxins, such as the barnase toxin or diphtheria toxin A (DTA). Both systems have been used in plants before to eliminate certain flower tissues, i.e., reproductive organs. In tobacco and *Arabidopsis*, the barnase system could be applied to induce female sterility, by targeting the stigmatic secretory zone of pistils, or male sterility, by targeting the mature stamen, respectively^25,26^. Similarly, using DTA in combination with the promoter of the floral homeotic protein AP3, expressed in petal and stamen tissue, the respective tissue could be eliminated and, thus, male sterility could be induced^27^. Next to toxins, also the UV laser ablation technique has been used successfully for cell elimination, i.e., in root endodermis cells to analyze wound response and regeneration processes^28,29^.

The major advantage of the CRISPR-Kill system is the broad range of applications. Besides tissue elimination, it can be applied for purposes not aiming for cell elimination but rather genome engineering. In contrast to the classical methods that eliminate cells in a rampant manner, the CRISPR-Kill system targets specific genomic sites and, thus, can be programmed to cleave individual chromosomes. So far, it has only been shown in mammals that individual chromosomes can be eliminated through the induction of multiple DSBs^30,31^. Transferring this to plants, CRISPR-Kill-based chromosome elimination might be an attractive application in plant hybrids, especially if coupled with chromosome restructuring^32,33^.

The CRISPR-Kill system is easy to adapt for use in other plant species, or even other multicellular eukaryotes, such as animals or fungi, due to the high conservation of the target regions. It will be interesting to combine it with an inducible and two-component system to control Cas9 expression, so that CRISPR-Kill can be controlled both temporally and spatially^22,34^.

CRISPR-Kill could also be an attractive tool for applications in synthetic biology. Cell-type specificity is a common characteristic of multiple biochemical pathways in plants, especially in secondary metabolism. Metabolites important for plant defense are often produced in specific cell types to avoid the peril of autotoxicity and to maximize the defense function^35^. Using the CRISPR-Kill system, the change of secondary metabolite composition within a plant and also the elimination or even production of new specific metabolites might become achievable. Thus, food production as well as pharmaceutical applications could benefit from this technology.

## Methods

### T-DNA constructs

DNA constructs were generated using the Gateway®-compatible (Thermo Fisher Scientific Inc., https://www.thermofisher.com) pDe-Sa-Cas9 and pEn-Sa-Chimera plasmids^14^. The kanamycin resistance cassette was exchanged with gentamycin using the restriction enzymes *PmeI* and *Sbf*I. The target sequences for break induction were ligated with pEn-Sa-Chimera as oligonucleotides, using AS1-AS12 (see Supplementary Table 1 for oligonucleotide sequences). Via Gateway LR reaction, the sgRNA expression cassette was transferred into the pDe-Sa-Cas9 vector, resulting in Ubi-SaCas9-ADH1, Ubi-SaCas9-IGS, Ubi-SaCas9-ITS1-1, Ubi-SaCas9-ITS1-2, Ubi-SaCas9-ITS2-1, and Ubi-SaCas9-Cen. The *AP1* promoter was amplified from *Arabidopsis* genomic DNA using the primer combination AS13 and AS14 (see Supplementary Table 2 for oligonucleotide sequences). The *XPP* promoter was synthesized (BioCat GmbH, https://www.biocat.com) and later amplified using the Primer combination AS15 and AS16 (see Supplementary Table 2 for oligonucleotide sequences). By *EcoR*I restriction enzyme digestion and Gibson Assembly® (New England Biolabs Inc., https://international.neb.com), the PcUbi4-2 of Ubi-SaCas9-ADH1 and Ubi-SaCas9-ITS2-1 and Ubi-SaCas9-Cen were exchanged with the *AP1* and *XPP* promoter to generate the tissue-specific constructs. For alignment and analysis of Sanger sequencing data, the software ApE was used. All constructs are available on request.

### Plant material and growth conditions

Experiments were performed in the *Arabidopsis thaliana* background Columbia-0 (Col-0). In addition, the T-DNA insertion lines *ku70-1* (*At1g16970*, SALK_123114)^36^, *lig4-5* (*At5g57160*, SALK_095962)^37^, and *rad54-1* (*At3g19210*, SALK_038057)^15^ were obtained from the SALK collection^38^. Plants were cultivated in the greenhouse on soil (1:1 mixture of Floraton [Floragard] and vermiculite [2–3 mm, Deutsche Vermiculite Dämmstoff]) with 16 h light (Phillips, Master, TL-D36W/840) and 8 h darkness at 22 °C. When sterile conditions were required, plants were cultured in a CLU-36L4 plant culture chamber (Percival Scientific) with stable conditions of 16 h light at 22 °C and 8 h dark at 20 °C on germination media (GM: 4.9 g/L Murashige and Skoog medium [Duchefa], 10 g/L Suc, and 7.6 g/L; pH 5.7 with potassium hydroxide). Seeds were surface-sterilized using 4% sodium hypochlorite and stratified overnight at 4 °C. For *Agrobacterium*-mediated plant transformation, floral-dipping was performed.

### Deep sequencing

For next-generation sequencing analyses (NGS) of the ADH1 locus, the genomic DNA of a pool of 40 primary transformants was extracted. To analyze repair patterns and SaCas9 efficiency, primers with 6 bp overhangs (AS17-AS20, see Supplementary Table 2 for oligonucleotide sequences) were designed to amplify a fragment containing the target site. For NGS analysis of the 45S rDNA ITS locus, DNA extraction of only the floral tissue was performed. Primer with 32 and 33 bp overhangs was designed (AS29 and AS30) for amplification of the target site. The 300–500 bp amplicons were generated using Q5 High-Fidelity DNA polymerase (New England Biolabs) and later purified using the peqGOLD Cycle Pure Kit (Peqlab). The amplicons were sequenced with the Illumina HiSeq platform at GATC Biotech. The quality of the reads was verified using the CLC Genomics Workbench (Qiagen Bioinformatics) and later analyzed using the CRISPR RGEN TOOL (Cas analyzer^39^) (http://www.rgenome.net/cas-analyzer/#!). Parameters were set for minimum frequency (*n*) = 1 and comparison range *R* = 200, no wild-type marker (*r*). Afterward, the data were analyzed using R Studio and Excel.

### Determination of T1 survival rate

T1 seeds were sown on GM containing the respective selection marker. After 14 days, the number of transgenic plants was counted and put in relation to the total seed number to determine the survival rate.

### Quantitative real-time PCR

To determine the amount of 45S rDNA copy numbers, real-time PCR analysis was conducted. Genomic DNA was extracted from 14-day-old *A. thaliana* seedlings and 0.5 ng of DNA were combined with 5 μL 2x qPCR Master Mix KAPA SYBR FAST (Sigma-Aldrich Chemie GmbH), 0.5 μL of forward and reverse primer (10 mM, AS21-AS28, see Supplementary Table 2 for oligonucleotide sequences) and filled up with distilled water to a total volume of 10 μL. The Light Cycler480 instrument (384-well block system; Roche Diagnostics) was used for analysis. All rDNA genes (18S, 5.8S, and 25S) as well as the reference gene Ubiquitin 10 were tested in three technical, as well as three biological replicates.

### Phenotypic analysis

To analyze phenotypical differences in the floral tissue, plants were grown in the greenhouse for 4 weeks. In total, 19 control plants, 133 pAP1-SaCas9-ITS2-1, and 40 pAP1-SaCas9-Cen lines were screened for phenotypical differences and sorted into different categories, dependening on the extent of these differences. Analysis was performed using a binocular microscope and pictures were taken with VisiCam 5 plus using the software IS Visicam Image analyser (VWR, https://www.vwr.com). To analyze phenotypical differences in the root, plants were first selected for transgene integration on selection medium containing PPT for 13 days. In total, 108 control plants of each line were transferred from selection medium to germination medium. After another 7 days, the number of lateral roots was determined. In addition, relative lateral root length, in relation to the main root length, was determined by the Image J add-on SmartRoot^40^. To analyze the effects of CRISPR-Kill in roots in the T2 generation, T1 lines were propagated to T2 and selected for heterozygous T-DNA integration on selection medium containing PPT. In total, three plants each out of 30 T2 lines were analyzed as described above.

### Statistical methods

Differences in survival rates were determined by applying a two-sided, two-sample *t*-test with unequal variance. For root number and relative lateral root length, a one-way ANOVA was performed: ns *P* > 0.05, *P ≤ 0.05; **P < 0.01; ***P < 0.001; ****P < 0.0001.

### Reporting summary

Further information on research design is available in the Nature Research Reporting Summary linked to this article.

## Supplementary information

Supplementary Information

Reporting Summary

## Data Availability

Deep sequencing data that support the findings of this study have been deposited in SRA [accession: PRJNA726366]. Source data are provided with this paper.

## Source data

Source Data

## Code availability

R code for detailed analysis of NGS data has been deposited to GitHub [https://github.com/PuchtaLab/CRISPR-Kill/blob/main/R-code].
