# Supplementary material for: Using CRISPR-Kill for organ specific cell elimination by cleavage of tandem repeats
Source: Nat Commun. 2022 Mar 21;13:1502. doi: 10.1038/s41467-022-29130-w (PMC8938420; doi:10.1038/s41467-022-29130-w)
Supplement: Supplementary file 1 — Supplementary Information [file 41467_2022_29130_MOESM1_ESM.pdf]

Using CRISPR-Kill for organ specific cell elimination by cleavage of tandem repeats

Schindele *et al.*

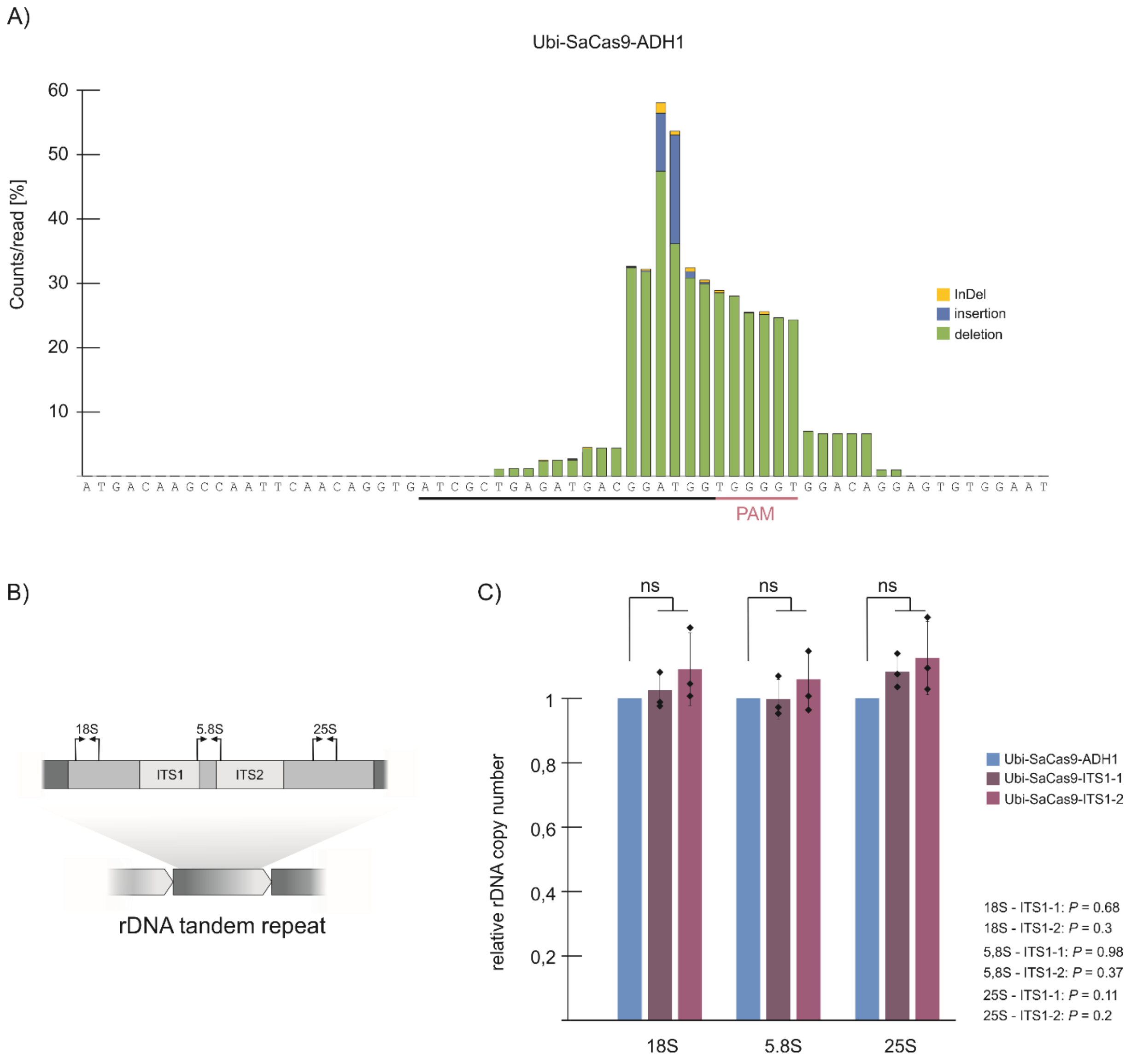

Supplementary Figure 1. Deep sequencing analysis of SaCas9-ADH1 lines and copy number analysis of the rDNA locus.

A) Distribution of mutations in Ubi-SaCas9-ADH1 plants of *A. thaliana*. Mutation frequencies of up to 60% could be observed. Most mutations were deletions (green), followed by substitutions (yellow) and insertions (blue) within the protospacer (underlined in grey) and upstream of the PAM sequence (underlined in red). **B)** Copy numbers of 18S, 5.8S and 25S rDNA sequences were analyzed using qRT-PCR with primer pairs for each functional subunit, as indicated in the schematic overview of one 45S rDNA unit. **C)** Copy numbers of 18S, 5.8S and 25S rDNA repeats were determined in T1 plants for each construct independently (n=3 biologically independent samples, consisting of three pooled independent T1 plants each). Data are presented as normalized mean values ± SD. No reduction in the 45S rDNA copy number could be observed in the rDNA cleavage lines, Ubi-SaCas9-ITS1-1 and Ubi-SaCas9-ITS1-2, compared to the control line, Ubi-SaCas9-ADH1. Statistical differences were calculated using a two-tailed *t*-test with unequal variances: ns = $P > 0.05$. Source data are provided as a Source Data file.

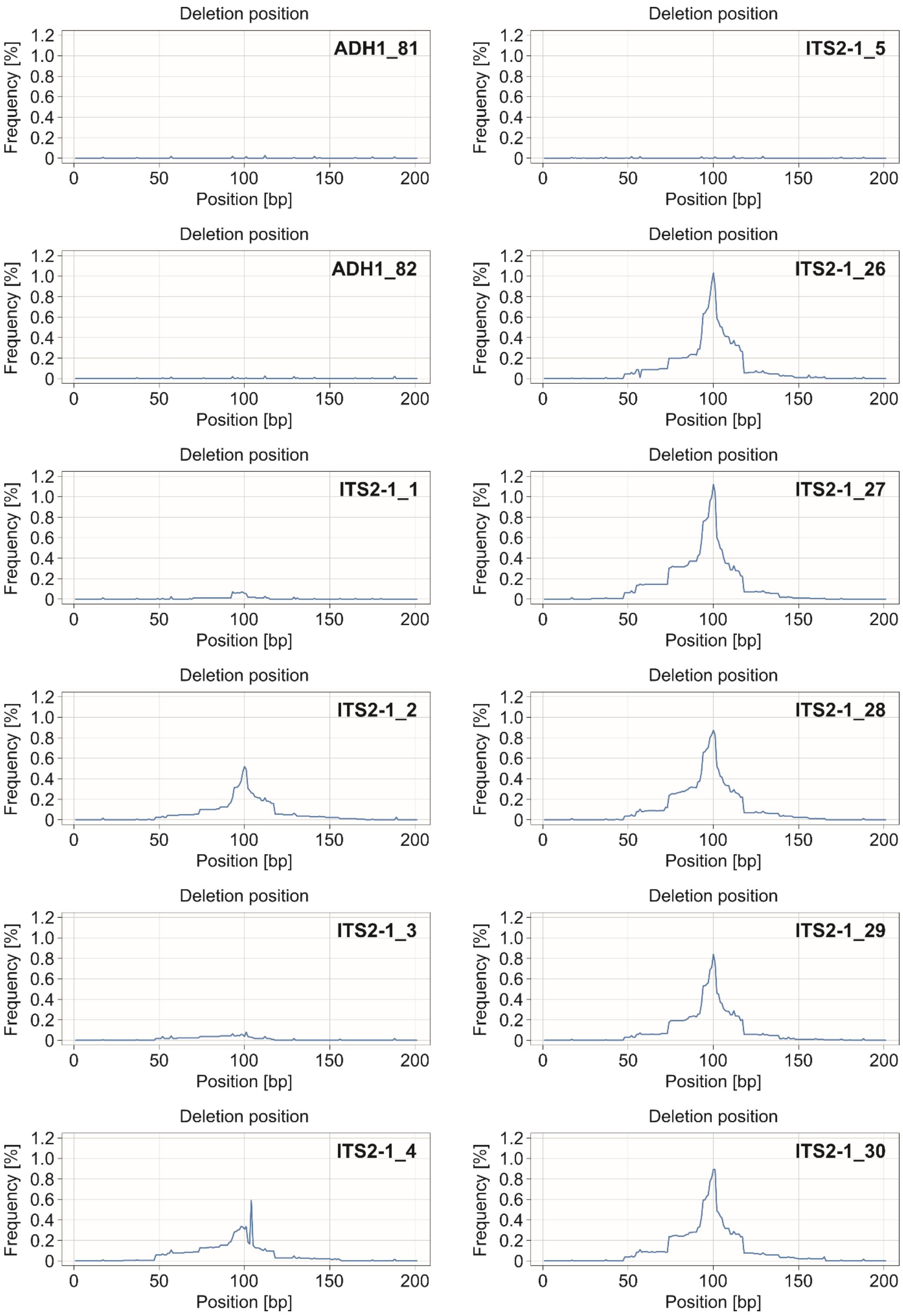

Supplementary Figure 2. Distribution of deletions at the ITS2-1 target site in 45S rDNA CRISPR-Kill and control lines. Distribution of deletions in two AP1-SaCas9-ADH1 lines and ten AP1-SaCas9-ITS2-1 lines. The distribution was determined at the ITS2-1 locus. Illumina sequencing data were evaluated using the Cas-Analyzer online tool. The cleavage is located at position 100 bp. The two ADH1 control lines (#81 and #82) showed no repair events at the target site, while nine out of ten CRISPR-Kill lines showed repair events correlating with the severity of the respective phenotype.

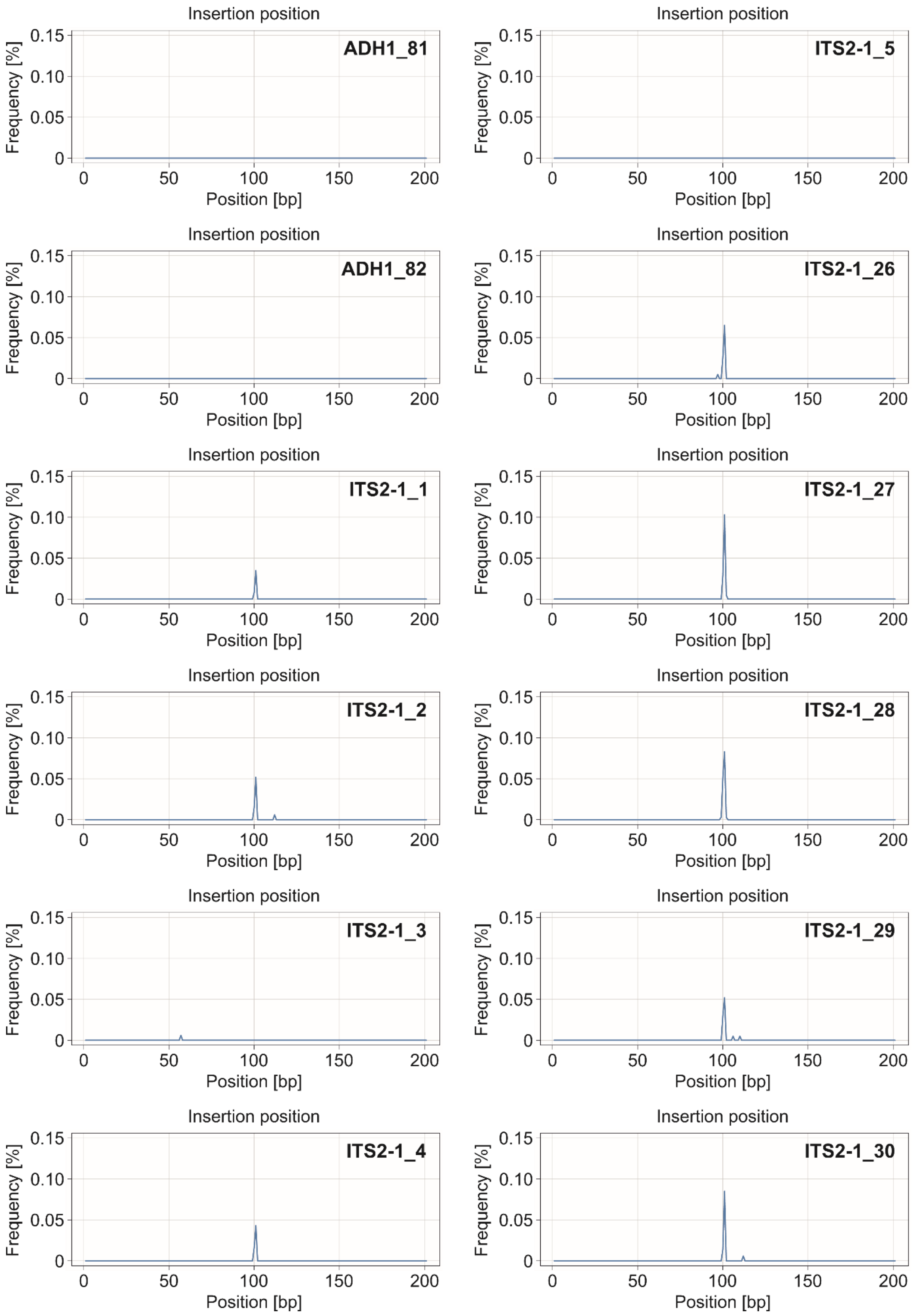

Supplementary Figure 3. Distribution of insertions at the ITS2-1 target site in 45S rDNA CRISPR-Kill and control lines. Distribution of insertions in two AP1-SaCas9-ADH1 lines and ten AP1-SaCas9-ITS2-1 lines. The distribution was determined at the ITS2-1 locus. Illumina sequencing data were evaluated using the Cas-Analyzer online tool. The cleavage is located at position 100 bp. The two ADH1 control lines (#81 and #82) showed no repair events at the target site, while eight out of ten CRISPR-Kill lines showed repair events correlating with the severity of the respective phenotype.

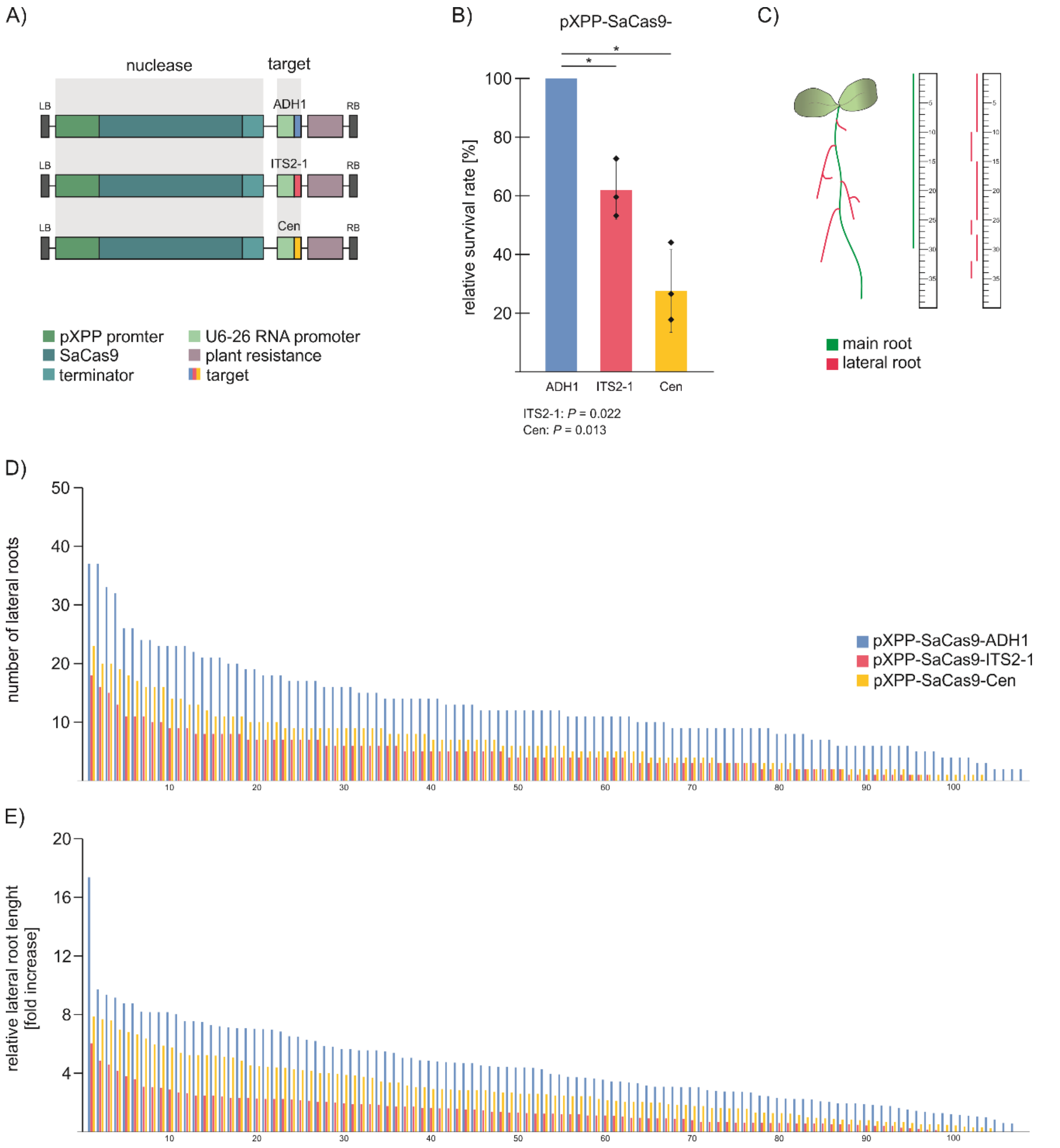

Supplementary Figure 4. Analysis of lateral root-specific pXPP-CRISPR-Kill lines.
A) Overview of constructs used in lateral root elimination. The nuclease SaCas9 (petrol) is under control of the XPP promoter (green) for expression in pericycle cells. The control construct (ADH1, blue) targets the *ADH1* gene. The 45S rDNA control construct (ITS2-1, red) targets the 45S rDNA and the centromeric construct (Cen, yellow) targets the centromere repeats. **B)** Relative survival rate of T1 plants. The tissue-specific expression of CRISPR-Kill constructs in the pericycle cells led to a slight reduction by 36% in the case of pXPP-SaCas9-ITS2-1 and a stronger reduction by 72% in the case of pXPP-SaCas9-Cen (n=3). Data are presented as normalized mean values ± SD. $P = 0.022$ for ITS2-1; $P = 0.013$ for Cen **C)** Schematic overview of relative lateral root determination. The length of the main root (green) as well as the sum of all lateral roots (red) of one T1 plant was determined. **D)** Distributions of lateral root number of single lines for pXPP-SaCas9-ADH1 (blue), pXPP-SaCas9-ITS2-1 (red) and pXPP-SaCas9-Cen (yellow). Each bar represents one T1 line. Lines were grouped based on their lateral root number in descending order. **E)** Distribution of sum of the length of all lateral roots in comparison to the main root length of single lines for pXPP-SaCas9-ADH1 (blue), pXPP-SaCas9-ITS2-1 (red) and pXPP-SaCas9-Cen (yellow), whereby each bar demonstrates a single T1 line. Lines were grouped based on their lateral root length in descending order. A comparison of single CRISPR-Kill lines (red and yellow) with the control line (blue) showed an explicit reduction in relative lateral root length. Statistical differences were calculated using a two-tailed *t*-test with unequal variances: * $p<0.05$. Source data are provided as a Source Data file.

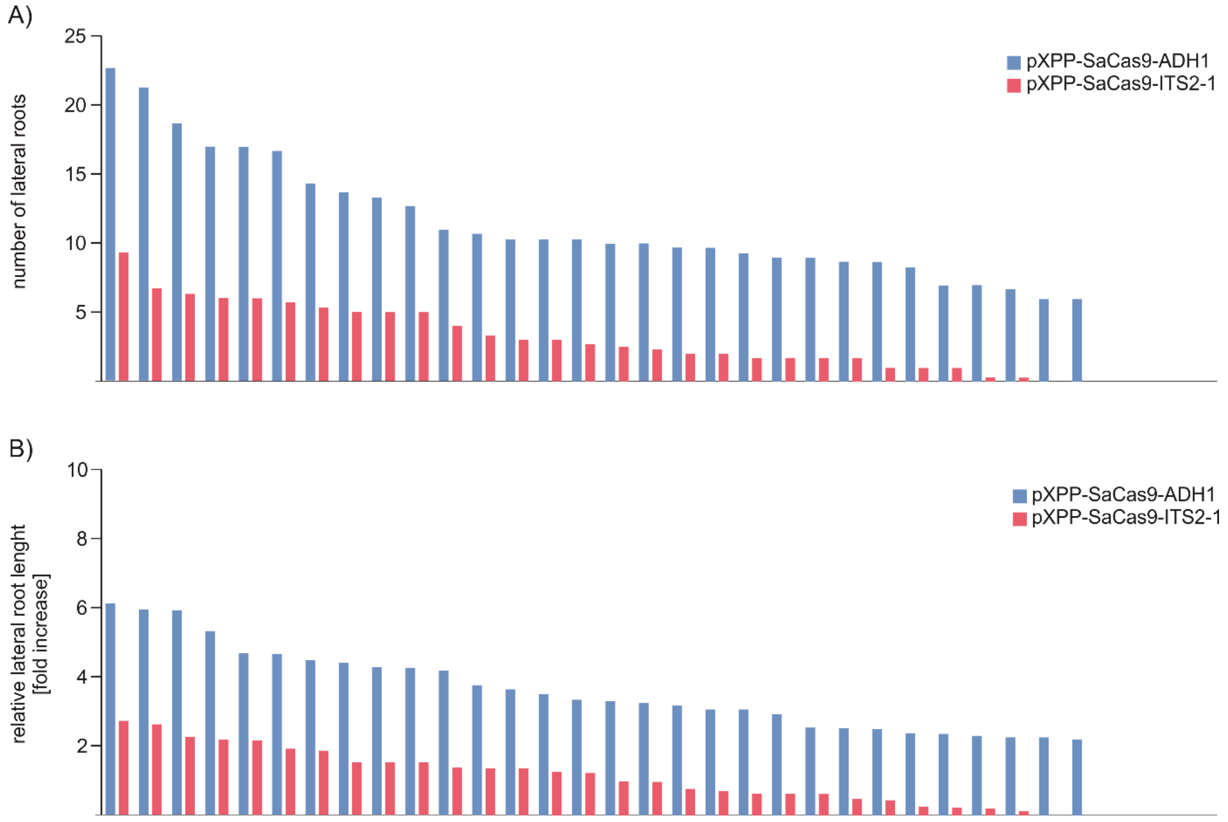

Supplementary Figure 5. Analysis of lateral root elimination in pXPP-SaCas9-ITS2-1 T2 lines.

A) Distribution of lateral root number of single lines for pXPP-SaCas9-ADH1 (blue) and pXPP-SaCas9-ITS2-1 (red) in the T2 generation. Lines were grouped based on their lateral root number in descending order. **B)** Distribution of sum of the length of all lateral roots in comparison to the main root length of single lines for pXPP-SaCas9-ADH1 (blue) and pXPP-SaCas9-ITS2-1 (red) in the T2 generation. Lines were grouped based on their lateral root length in descending order. Comparison of the single 45S rDNA CRISPR-Kill lines (red) with the control line (blue) showed a very clear reduction in relative lateral root length.

Supplementary Table 1. Oligonucleotides for spacer sequences for the cloning of T-DNA constructs.

Target site	No.	Name	Sequence 5'-3'
ADH1	AS1	SaCas9-ADH1-FW	attgATCGCTGAGATGACGGATGG
	AS2	SaCas9-ADH1-RV	aaacCCATCCGTCATCTCAGCGAT
IGS	AS3	SaCas9-IGS-FW	attgCCCAAGTCCGAACCGAAAAA
	AS4	SaCas9-IGS-RV	aaacTTTTTCGGTTCGGACTTGGG
ITS1-1	AS5	SaCas9-ITS1-1-FW	attgAGAGTGGTGATCTTTGGTTC
	AS6	SaCas9-ITS1-1-RV	aaacGAACCAAAGATCACCACTCT
ITS1-2	AS7	SaCas9-ITS1-2-FW	attgTTCCTTGACACTTTTCGTGC
	AS8	SaCas9-ITS1-2- RV	aaacGCACGAAAAGTGTCAAGGAA
ITS2-1	AS9	SaCas9-ITS2-1-FW	attgCTGGGGTCGCTATATGGACT
	AS10	SaCas9-ITS2-1- RV	aaacAGTCCATATAGCGACCCCAG
Cen	AS11	SaCas9-Cen-FW	attgTTGGCTTTGTGTCTTCTAAC
	AS12	SaCas9-Cen- RV	aaacGTTAGAAGACACAAAGCCAA

Supplementary Table 2. Primer sequences used in this study.

No.	Name	Sequence 5'-3'
		Primers used in promoter cloning
AS13	pAP1-FW	TATGACATGATTACGAATTCTGGAACCAAAGCTTAATG
AS14	pAP1-RV	CTTCATGGCGCGCCGAATTCTTTTGATCCTTTTTTAAGAAAC
AS15	pXPP-FW	TATGACATGATTACGAATTCGGTACCGTGTTTGGTTCG
AS16	pXPP-RV	CTTCATGGCGCGCCGAATTCGGAAATCTTCGTGTGTTAAG
		Primers used for *Next Generation Sequencing*
AS17	NGS-Sa-ADH1-FW	TGTGAACCCGAAAGACCATG
AS18	NGS-Sa-ADH1-RV	TCCGAATGATGTGAAGGGAG
AS19	NGS-Sa-IGS-FW	TTGGCACCGGTGTCTCC
AS20	NGS-Sa-IGS-FW	GGCCTCGAAGAATCCATGTCA
AS29	NGS-Sa-ITS2-FW	ACACTCTTTCCCTACACGACGCTCTTCCGATCTCTTCTGGCCGAGGGC
AS30	NGS-Sa-ITS2-FW	GACTGGAGTTCAGACGTGTGCTCTTCCGATCTCTCGTAGACAGCGCCTCG
		Primers used in qPCR
AS21	UBQ10-FW	AACGGGAAAGACGATTAC
AS22	UBQ10-RV	ACAAGATGAAGGGTGGAC
AS23	18S (XbaI)-FW	CTAGAGCTAATACGTGCAACAAAC
AS24	18S (HpaI)-RV	GAATCGAACCCTAATTCTCCG
AS25	5,8S-FW	CGGAGTGTGGGCGGATG
AS26	5,8S-RV	GTGAGGGACGACGATTTG
AS27	25S-FW	GTGCGAGTCAACGGGTG
AS28	25S-RV	ACCCAAGTCAGACGAACG
