# Supplementary material for: Using CRISPR-Kill for organ specific cell elimination by cleavage of tandem repeats
Source: Nat Commun. 2022 Mar 21;13:1502. doi: 10.1038/s41467-022-29130-w (PMC8938420; doi:10.1038/s41467-022-29130-w)
Supplement: Supplementary file 2 — Reporting Summary [file 41467_2022_29130_MOESM2_ESM.pdf]

nature portfolio

Corresponding author(s): Holger Puchta
Last updated by author(s): Feb 28, 2022

Reporting Summary

Nature Portfolio wishes to improve the reproducibility of the work that we publish. This form provides structure for consistency and transparency in reporting. For further information on Nature Portfolio policies, see our Editorial Policies and the Editorial Policy Checklist.

Statistics

For all statistical analyses, confirm that the following items are present in the figure legend, table legend, main text, or Methods section.

n/a	Confirmed	
☐	☒	The exact sample size (*n*) for each experimental group/condition, given as a discrete number and unit of measurement
☐	☒	A statement on whether measurements were taken from distinct samples or whether the same sample was measured repeatedly
☐	☒	The statistical test(s) used AND whether they are one- or two-sided<br>*Only common tests should be described solely by name; describe more complex techniques in the Methods section.*
☒	☐	A description of all covariates tested
☒	☐	A description of any assumptions or corrections, such as tests of normality and adjustment for multiple comparisons
☐	☒	A full description of the statistical parameters including central tendency (e.g. means) or other basic estimates (e.g. regression coefficient) AND variation (e.g. standard deviation) or associated estimates of uncertainty (e.g. confidence intervals)
☐	☒	For null hypothesis testing, the test statistic (e.g. *F*, *t*, *r*) with confidence intervals, effect sizes, degrees of freedom and *P* value noted<br>*Give P values as exact values whenever suitable.*
☒	☐	For Bayesian analysis, information on the choice of priors and Markov chain Monte Carlo settings
☒	☐	For hierarchical and complex designs, identification of the appropriate level for tests and full reporting of outcomes
☒	☐	Estimates of effect sizes (e.g. Cohen's *d*, Pearson's *r*), indicating how they were calculated

Our web collection on statistics for biologists contains articles on many of the points above.

Software and code

Policy information about availability of computer code

Data collection	No custom code was used in data collection. Sanger sequencing (SupremeRun tube) and next generation sequencing (NGSelect Amplikon 2nd PCR, Illumina) was performed by Eurofins Genomics. A binocular microscope (SZB300, VWR) equipped with the VisiCam 5 plus camera (VWR) and the software IS Visicam Image analyser (V3.9) was used. Please see material and methodes for details.
Data analysis	During the analysis we used published or open source software as well as commercial software. For evaluation of qPCR results the software LightCycler 480 SW 1.5.1 (Roche) was used. Raw reads were processed through CLC Genomics workbench (version 10.1.11). Subsequent analysis was done with the online tool CRISPR RGEN TOOL (Cas analyser, Park J et al., 2017) (http://www.rgenome.net/cas-analyzer/#!) followed by Rstudio (version 1.3.1073) with our own custom code which is available on GitHub (https://github.com/PuchtaLab/CRISPR-Kill/blob/main/R-code). Graphs were made in Rstudio (1.3.1073) for boxplot and Excel 2016 for bar charts. ApE (v2.0.55) was used for alignment and analysis of Sanger sequencing data. Root length was determined by the Image J (v1.8.0_172) add-on SmartRoot (Lobet et al., 2011).

For manuscripts utilizing custom algorithms or software that are central to the research but not yet described in published literature, software must be made available to editors and reviewers. We strongly encourage code deposition in a community repository (e.g. GitHub). See the Nature Portfolio guidelines for submitting code & software for further information.

Data

Policy information about availability of data

All manuscripts must include a data availability statement. This statement should provide the following information, where applicable:

- Accession codes, unique identifiers, or web links for publicly available datasets
- A description of any restrictions on data availability
- For clinical datasets or third party data, please ensure that the statement adheres to our policy

The authors declare that the data generated in this study are provided in the Supplementary Information/Source Data file. Source data are provided with this paper. Deep sequencing data that support the findings of this study have been deposited in SRA [accession: PRJNA726366] (https://www.ncbi.nlm.nih.gov/bioproject/PRJNA726366).

Field-specific reporting

Please select the one below that is the best fit for your research. If you are not sure, read the appropriate sections before making your selection.

- [x] Life sciences
- [ ] Behavioural & social sciences
- [ ] Ecological, evolutionary & environmental sciences

For a reference copy of the document with all sections, see nature.com/documents/nr-reporting-summary-flat.pdf

Life sciences study design

All studies must disclose on these points even when the disclosure is negative.

Sample size	No sample-size calculation was performed. To randomize bias from integration of the Cas9 nuclease, rather large sample sizes were chosen. For survival rate analysis, by default we prepare 10 large selection plates per line, which corresponds to at least 5000 seeds in each biologically independent experiment. For deep sequencing analysis of IGS-lines, 40 plants per line were pooled for DNA extraction. For flower phenotype analysis, 19 independent T1 control plants were used and at least 40 independent T1 CRISPR-Kill plants to cover the expected phenotypical range. For root analysis, 108 independent T1 lines as well as 3 plants each out of 30 T2 lines were analysed. According to our previous experience in the Arabidopsis system, all sample sizes are sufficient to ensure reproducibility and detect significant differences.
Data exclusions	No data has been excluded
Replication	All attempts at replication were successful. True biological replicates (i.e., independent plants) were used as replicates for statistical analyses. The number of replicates is given in the Figure legends. For deep sequencing, the experiment was performed only once with one biological replicate out of genotyping costs. To ensure reproducibility, the plants were grown in a growth chamber under well defined conditions and samples were taken at the same developmental stage.
Randomization	Plants were transformed with the respective constructs. T1 seeds of plants transformed with the same constructs were put on 10 large plates with selection medium and transgenic lines were randomly picked, equally distributed over all 10 plates. T2 analysis was performed with 30 T2 lines selected for heterozygous T-DNA integration. For deep sequencing analysis of single flowers (linking phenotype with genotype), 10 plants were picked, covering the complete phenotypical range.
Blinding	Not required for most analyses, as samples were processed identically through standard and in some cases automated procedures (survival rate, qPCR, deep sequencing) that should not bias outcomes.<br>Phenotypical analysis is done blinded by randomly numbering the plant lines and assigning the genotype after analysis.

Reporting for specific materials, systems and methods

We require information from authors about some types of materials, experimental systems and methods used in many studies. Here, indicate whether each material, system or method listed is relevant to your study. If you are not sure if a list item applies to your research, read the appropriate section before selecting a response.

Materials & experimental systems

n/a	Involved in the study	
[x]	[ ]	Antibodies
[x]	[ ]	Eukaryotic cell lines
[x]	[ ]	Palaeontology and archaeology
[x]	[ ]	Animals and other organisms
[x]	[ ]	Human research participants
[x]	[ ]	Clinical data
[x]	[ ]	Dual use research of concern

Methods

n/a	Involved in the study	
[x]	[ ]	ChIP-seq
[x]	[ ]	Flow cytometry
[x]	[ ]	MRI-based neuroimaging
